# Recognition of allergic conjunctivitis in patients with allergic rhinitis

**DOI:** 10.1186/1939-4551-6-4

**Published:** 2013-02-12

**Authors:** Daniel C Williams, Gabrielle Edney, Bianca Maiden, Peter K Smith

**Affiliations:** 1 Department, Institute, School of Medicine Gold Coast Campus, Griffith University, 16 High Street, Gold Coast 4222, Australia

## Abstract

**Aims:**

To identify the incidence of allergic conjunctivitis in patients with allergic rhinitis.

**Methods:**

One hundred and eighty seven consecutive patients with allergic rhinitis (AR) were directly questioned if they have allergic conjunctivitis (AC) and this was clarified using standard screening questions relating to red, itchy and watery eyes recorded through a total ocular symptom score (TOSS). Patients were also asked about further symptoms that may be attributable to AC: eyelid dermatitis, frequent blinking; eye sensitivity and frontal headache from squinting or. blinking. All patients were given a drop of olopatadine hydrochloride 0.1% in each eye to help identify “silent” disease. 20 healthy non-atopic controls were also treated with olopatadine drops and questioned on ocular symptoms.

**Results:**

Fifty five percent of patients with AR were identified as having AC by direct questioning and the use of the TOSS questionaire. A further 41% were identifiable by asking additional questions and performing therapeutic challenge with olopadatine.

**Conclusions:**

AC is a frequent comorbid condition occurring in 95% of our patients with AR. Only 55% of patients were able to identify that they had AC based on standard screening questions. Additional specific questioning and a therapeutic challenge in suspected patients can help identify patients who may benefit from treatment of AC.

## Background

Allergic conjunctivitis (AC) commonly manifests as itchy, watering or red eyes, which comprise the symptoms of the total ocular symptom scores (TOSS) [1-3].

The incidence of AC in developed countries is 20% [4-6] with a high co-morbidity of allergic rhinitis (AR) [5,6]. Recognition of AC is unreported even in patients with recognised AR [6,7]. Under-recognition of AR is common, with the proportion of undiagnosed AR patients ranging from 25–60% [8].

Clinically, it is apparent that AC patients have heightened sensitivity, tending to blink and squint more, contributing to frontal tension headaches. Rubbing of eyelids can contribute to dermatitis, with patients focusing more on the dermatitis than conjunctival symptoms.

Olopatadine hydrochloride 0.1% was selected for its efficacy in AC, providing negligible side effect profile and rapid onset of action, evident from five minutes post administration [9-12].

### Objective

Identify the incidence of AC in patients with AR.

## Methods

One hundred and eighty seven consecutive patients – paediatrics and adults, during pollen season, with Allergist diagnosed AR attending an outpatient clinic in Southport, on the Gold Coast, were directly questioned if they have AC, clarified by using standard screening questions of red, itchy and watery eyes and quantified by TOSS. Patients were asked about indirect symptoms that may be attributable to AC: eyelid dermatitis, frequent blinking, eye sensitivity and frontal headache. Patients were given a drop of Olopatadine in each eye to help identify “silent” disease.

Patients were prospectively diagnosed with allergic rhinitis based on clinical history, examination and concurrent skin prick testing by an allergy specialist. Patients were instructed not to take antihistamines for at least 48 hours prior to assessment.

Twenty controls without a clinical history of AR or AC were also treated with olopatadine drops to determine if there was a non-specific lubricating effect of olopatadine hydrochloride.

We did not note history of lasik surgery or pterygia in any of our patients, nor were any of our participants wearing contacts at the time of olopatadine administration.

Ethics approval was obtained from Griffith University Human Research Ethics Committee.

This trial was registered with Australia New Zealand Clinical Trials Registry (ANZCTR).

Data was analyzed using Microsoft Excel data-base software (Microsoft Corp., Redlands, CA) using a paired t-test for pre and post TOSS with Olopatadine challenge. A Pearson correlation coefficient was used to compare relationship between TOSS positivity and presence of additional symptoms and the presence of symptoms and response to a therapeutic challenge with olopatadine

## Results

Fifty three percent of patients identified themselves as having AC on direct questioning and enquiring about specific TOSS symptoms. Additional possible symptoms attributable to AC were squint 51%, blinking 52%, frontal headache 60% and eyelid dermatitis 45%.

Olopatadine reduced TOSS scores within 5 minutes of treatment (1.34 +/− 1.66 vs 0.486 +/− 0.83. p < 0.01). 146 (78.1%) subjects noted improvement verses 41 (21.9%) noting no change in ocular symptoms. Based on a negative history of AC and baseline TOSS of 0, therapeutic challenge of Olopatadine identified 77 (41.2%) silent sufferers of AC.

No effect on TOSS was observed in control patients treated with olopatadine.

One hundred and sixty six (88.8%) AC subjects were identified through TOSS symptoms. Plausible indirect AC symptoms detected 158 (84.5%) subjects. However, combining standard TOSS and additional questions detected 177 (94.7%) AC subjects. Presence of additional ocular symptoms correlated both with TOSS responses and beneficial effect of a therapeutic challenge with Olopatadine (r = 0.60 , P < 0.05).

## Discussion

AC was identified in 53.5% of patients with AR using direct questioning in relation to history of AC. This is comparable with other studies [6,7,13-18]. Specific questioning regarding indirect symptoms increased the incidence of AC in patients with AR to 94.7%. Olopatadine therapeutic challenge was performed in 20 healthy, non-atopic controls to ensure there was no non-specific lubricating effect of the eye drop.

As this was a therapeutic challenge, a lubricant could have improved symptoms and it would most likely help patients with allergic conjunctivitis. There was no improvement in controls that were given this lubricant effect and hence TOSS improvement can be attributed to olopatadine, not to non-specific lubricating effect.

The co-existence of AC is well recognised in patients with AR [6,7,13-18] although co-reporting frequency may be as low as 40%. Under-recognition of allergic conjunctivitis may be due to patients and physicians paying more attention to allergic comorbidities such as AR or rhinitis or the under-appreciation of the variability of eye symptoms in patients with AC [6,19-21].

Our study confirms patients have an under-appreciation of symptoms, even when prompted with specific questions, and the value of a therapeutic challenge. Our data suggests approximately 40% of AR patients felt their symptoms represented as normal. This is significant because the presence and lack of treatment of AC contributes negatively to their quality of life [22].

Limitations of this study include; it was an open clinical audit and direct survey questions were used. Questions of children were occasionally influenced by their parent’s answers or prompting. Headache is a symptom associated with AR [3] and may not localise towards ocular symptoms. However we did find an association with a history of headache and response to a therapeutic challenge.

## Conclusion

In conclusion, the standard AC screening questions identified just over half of the patients with AC. As suggested by others, the absence of a history does not negate the value of examining the conjunctiva. Additionally we suggest that symptoms of blinking, squinting, eyelid dermatitis and frontal headache and use of olopatadine hydrochloride eye drops can help identify patients with “silent” symptoms.

## Consent

Written informed consent was obtained from all the patients for publication of this report and any accompanying images.

## Competing interests

The authors declare that they have no competing interests.

## Authors’ contributions

DW, GE, BM and PS carried out data collection. DW and GE collated and analysed statistics. DW drafted the manuscript. PS finalized the manuscript. All authors read and approved the final manuscript.
